# Cross-cancer transcriptomic analysis reveals conserved developmental, ferroptosis, and immune remodeling modules in Wilms tumor and oral cancer

**DOI:** 10.3389/fgene.2026.1861806

**Published:** 2026-08-17

**Authors:** Guijun Li, Jinhong Bai, Sai Hu, Xiuli Gao, Fang Wang, Song Li

**Affiliations:** 1 Department of Pediatric Surgery, Peking University First Hospital Ningxia Women and Children’s Hospital (Ningxia Hui Autonomous Region Maternal and Child Health Hospital), The Third Clinical Medical College of Ningxia Medical University, Yinchuan, Ningxia, China; 2 Department of Stoma, Peking University First Hospital, Beijing, China; 3 Department of Radiation Therapy, Affiliated Hospital of Hebei University, Baoding, Hebei, China; 4 Department of Stomatology, Affiliated Hospital of Hebei University, Baoding, Hebei, China

**Keywords:** cross-cancer analysis, ferroptosis, iron metabolism, nephroblastoma, oral cancer, transcriptomic module, tumor immune microenvironment, Wilms tumor

## Abstract

**Background:**

Wilms tumor (nephroblastoma) is a pediatric renal malignancy driven by disrupted nephrogenesis, yet its transcriptomic relationship with ferroptosis and immune remodeling remains poorly characterized.

**Methods:**

We analyzed GSE66405 microarray data (28 Wilms tumor samples) together with an oral cancer 10X single-cell reference dataset to evaluate six biologically defined modules: developmental/nephrogenesis, Wnt/beta-catenin, ferroptosis/iron metabolism, immune/inflammatory, extracellular matrix, and cell-cycle. Module scores were calculated as mean z-scores across curated gene sets, and inter-module Pearson correlations, co-expression network analyses, and unsupervised clustering were performed in Python using scipy, scikit-learn, networkx, and matplotlib.

**Results:**

Developmental and Wnt programs formed the central transcriptomic axis of Wilms tumor, with WT1, SIX1, SIX2, PAX2, and CTNNB1 as hub genes (Pearson r = 0.868 between module scores, P < 0.001). The developmental module showed the strongest correlation with ferroptosis (r = 0.920, P < 0.001), while ferroptosis and immune modules were also tightly linked (r = 0.851, P < 0.001). Ferroptosis-related genes (HMOX1, FTL, SLC40A1, TFRC, GPX4, SLC7A11) and immune markers (CD68, CD163, CD8A, GZMB) were detected as secondary remodeling modules associated with the developmental core. Cross-cancer comparison revealed partial conservation of ferroptosis and immune module architecture in the oral cancer reference, whereas the developmental-Wnt axis remained Wilms tumor-specific. *In vitro* qRT-PCR validation in WiT49 (Wilms tumor) and CAL-27 (oral squamous cell carcinoma) cell lines confirmed differential ferroptosis gene expression: HMOX1 and TFRC were significantly upregulated in WiT49 cells (p < 0.001), while GPX4 and SLC7A11 were relatively higher in CAL-27 cells (p < 0.01), corroborating lineage-specific iron metabolism remodeling.

**Conclusion:**

These results link developmental dysregulation, iron metabolism, and immune remodeling in Wilms tumor and identify candidate modules for future functional validation. The developmental-Wnt axis constitutes a Wilms tumor-specific transcriptomic core, while ferroptosis and immune programs represent partially conserved stress-response modules that may offer cross-lineage therapeutic relevance. *In vitro* qRT-PCR experiments in WiT49 and CAL-27 cells corroborated these transcriptomic findings, confirming lineage-differential expression of core ferroptosis regulators across the two cancer contexts.

## Introduction

Wilms tumor (nephroblastoma) is the most common pediatric renal malignancy, accounting for approximately 95% of kidney tumors in children and representing roughly 6% of all pediatric cancers, with an annual incidence of approximately one in 10,000 children under 15 years of age (Leslie et al., 2023; Rivera and Haber, 2005). The tumor occurs predominantly in children aged 3–4 years, is bilateral in approximately 5%–10% of cases, and is associated with a range of congenital syndromes including WAGR syndrome, Beckwith-Wiedemann syndrome, and Denys-Drash syndrome, underscoring its deep developmental origins (Leslie et al., 2023). Unlike adult renal carcinomas, which are predominantly driven by acquired somatic mutations in VHL, PBRM1, or mTOR pathway genes, Wilms tumor arises from aberrant kidney development characterized by persistent nephrogenic rests and disrupted mesenchymal-to-epithelial transition (Leslie et al., 2023; Rivera and Haber, 2005). The tumor is fundamentally a developmental disorder, with recurrent alterations in WT1, CTNNB1 (beta-catenin), SIX1/SIX2, and the DROSHA/DGCR8 miRNA processing complex identified across large-scale genomic studies (Gadd et al., 2017). WT1 mutations occur in approximately 10%–20% of Wilms tumor cases and frequently co-occur with CTNNB1 mutations in a non-random pattern, while SIX1/SIX2 hotspot mutations preferentially associate with diffuse anaplasia and high-risk blastemal histology (Gadd et al., 2017). Beyond individual gene alterations, the broader transcriptomic architecture of Wilms tumor recapitulates features of nephrogenesis, including activation of embryonic transcription factors and disruption of normal differentiation hierarchies (Rivera and Haber, 2005). This developmental basis provides a unique opportunity to examine how oncogenic transformation intersects with tissue-specific differentiation programs and metabolic adaptations of the developing kidney.

Ferroptosis, an iron-dependent form of regulated cell death driven by lipid peroxidation and executed through glutathione peroxidase 4 (GPX4) inactivation, has emerged as a critical stress-response pathway in cancer biology (Dixon et al., 2012; Stockwell et al., 2017). Unlike apoptosis or necroptosis, ferroptosis is mechanistically defined by iron-catalyzed oxidative damage to polyunsaturated fatty acid-containing phospholipids, resulting in membrane rupture and non-apoptotic cell death (Dixon et al., 2012). Key regulators include GPX4, the cystine-glutamate antiporter SLC7A11, the iron storage proteins FTH1 and FTL, and the transferrin receptor TFRC, which collectively govern cellular iron homeostasis and the capacity to neutralize lipid peroxides (Jiang et al., 2021). Additional modulators include NCOA4-mediated ferritinophagy, ACSL4-dependent phospholipid remodeling, and HMOX1-driven heme catabolism, which together determine the size of the labile iron pool available for Fenton chemistry and ultimately set cellular ferroptosis sensitivity. The master antioxidant transcription factor NFE2L2 (NRF2) provides a counterbalancing cytoprotective layer by transcriptionally upregulating multiple ferroptosis-suppressive targets including FTH1, SLC7A11, and HMOX1 itself. In pediatric solid tumors, ferroptosis susceptibility may intersect with developmental metabolic states, as proliferating embryonal cells maintain elevated iron requirements for DNA synthesis, mitochondrial respiration, and cofactor biosynthesis; however, systematic analysis of ferroptosis-related transcriptional programs in Wilms tumor has remained limited, and the relationship between iron metabolism dysregulation and the developmental core of this disease has not been characterized.

The tumor immune microenvironment (TIME) in Wilms tumor is substantially less characterized than in adult carcinomas such as clear cell renal cell carcinoma or hepatocellular carcinoma, despite accumulating evidence that immune infiltration patterns play clinically relevant roles in disease progression and outcome (Li et al., 2022). Macrophage infiltration, cytotoxic T-cell signatures, and inflammatory cytokine profiles have been documented in Wilms tumor tissue, with tumor-associated macrophages (TAMs), particularly the alternatively activated M2 phenotype expressing CD163 and MRC1, identified as the dominant immune infiltrating population and associated with advanced staging and unfavorable histology (Wang et al., 2024). Cytotoxic CD8^+^ T cells expressing GZMB and PRF1 are also detectable within the tumor microenvironment, though their functional states, spatial distribution, and contribution to clinical outcomes remain incompletely characterized. A further complexity is that Wilms tumor primary cells appear capable of actively shaping the immunological milieu, impairing NK cell cytotoxicity and polarizing monocytes toward an M2 macrophage phenotype to establish an immunosuppressive microenvironment (Fiore et al., 2021). Crosstalk between ferroptosis and immunity is increasingly recognized as a bidirectional regulatory axis: CD8^+^ T cells can promote tumor ferroptosis through interferon-gamma-mediated suppression of SLC7A11 and SLC3A2 expression, while iron metabolism reciprocally modulates macrophage polarization, with iron availability influencing the balance between pro-inflammatory M1 and immunosuppressive M2 macrophage states (Wang et al., 2019; Zhang et al., 2025). These overlapping regulatory networks suggest that ferroptosis and immune programs may be co-regulated in the Wilms tumor microenvironment, a hypothesis that warrants systematic transcriptomic evaluation.

Cross-cancer transcriptomic comparison has emerged as a powerful strategy to distinguish disease-specific programs from shared tumor-associated stress-response modules, and to identify conserved biological vulnerabilities that may be therapeutically exploitable across tumor types (Puram et al., 2017). Such analyses have revealed that certain metabolic and immune remodeling programs, including glycolytic reprogramming, hypoxia adaptation, and TGF-beta-driven immune exclusion, operate across diverse tumor lineages through conserved molecular mechanisms, whereas developmental and lineage-specific transcription factor programs tend to remain cancer-type-restricted. Oral squamous cell carcinoma (OSCC), arising from the stratified squamous epithelium of the oral cavity and oropharynx, is biologically distinct from Wilms tumor in both tissue of origin and developmental context; nevertheless, OSCC shares broad epithelial tumor characteristics including dysregulated cell adhesion, extracellular matrix remodeling, and inflammatory microenvironment composition, and has been extensively profiled at single-cell resolution using 10X Genomics platforms (Puram et al., 2017). Single-cell analyses of OSCC have characterized ferroptosis activity primarily within malignant epithelial compartments and identified ferroptosis-related prognostic gene signatures correlated with immune cell infiltration patterns (Tang et al., 2024). The availability of high-quality single-cell OSCC reference data, combined with its well-characterized ferroptosis and immune transcriptomic landscape, makes it a suitable external epithelial tumor reference with which to assess whether ferroptosis and immune remodeling modules observed in Wilms tumor are disease-specific features or represent broadly shared tumor stress-response programs that transcend lineage boundaries.

In this study, we applied a module-based transcriptomic framework to systematically characterize the molecular architecture of Wilms tumor and to assess cross-cancer conservation of key biological programs. Specifically, we: (1) characterized Wilms tumor transcriptomic architecture through six biologically defined modules spanning developmental/nephrogenesis, Wnt/beta-catenin, ferroptosis/iron metabolism, immune/inflammatory, extracellular matrix, and cell-cycle programs, using the publicly available GSE66405 microarray dataset comprising 32 Wilms tumor samples; (2) determined whether developmental and Wnt programs constitute the dominant molecular axis of Wilms tumor by evaluating inter-module correlation strength, co-expression network topology, and hub gene connectivity across all 32 samples; (3) assessed ferroptosis and immune signatures as secondary remodeling programs and examined their co-regulation with the developmental core at both module-score and gene-network levels; and (4) evaluated cross-cancer module conservation by comparing Wilms tumor module scores against an oral squamous cell carcinoma single-cell RNA-seq reference dataset, with the aim of identifying which modules are Wilms tumor-specific versus broadly conserved across distinct epithelial tumor contexts, thereby providing a foundation for future functional and mechanistic investigations of developmental dysregulation, iron metabolism, and immune remodeling in pediatric renal cancer.

## Methods

### Data sources

Wilms tumor expression data were obtained from the Gene Expression Omnibus (GEO) under accession GSE66405, comprising Agilent SurePrint G3 Human Gene Expression 8 × 60K Microarray raw TXT files from 28 Wilms tumor patient samples. Raw probe-level intensities were background-corrected using the normexp method, log2-transformed, and quantile-normalized across all samples to achieve comparable signal distributions. A total of 42,947 probe-level features passed quality filtering criteria based on detection confidence scores provided in the Agilent raw output files; probes flagged as non-detectable in more than 50% of samples were excluded prior to downstream analysis. Probes were mapped to HGNC-approved gene symbols using the Agilent platform annotation files (GPL13497), and where multiple probes mapped to the same gene symbol, the probe with the highest median expression across all 32 samples was selected as the representative value for that gene, yielding a non-redundant gene-level expression matrix for module scoring and network analyses. The oral cancer external epithelial tumor reference dataset (GSM5258385_SYSMH1) was a 10X Genomics Chromium single-cell RNA-seq barcode-feature-matrix derived from a fresh oral squamous cell carcinoma tumor specimen and deposited as part of a published single-cell landscape study. Both datasets are publicly available in de-identified form through GEO and did not require additional institutional ethics review.

### Oral cancer reference processing

The 10X Genomics sparse matrix (barcodes, features, matrix files) was imported using scanpy (v1.9) and subjected to standard quality control filtering. Cells were excluded if they contained fewer than 200 detected genes, if the proportion of counts mapped to mitochondrial genes exceeded 20%, or if total unique molecular identifier (UMI) counts fell below the 5th or above the 95th percentile of the sample distribution; these thresholds were applied to remove low-quality cells, potential empty droplets, and putative doublets or ambient RNA-contaminated barcodes. Following quality control, the filtered matrix was library-size normalized by scaling each cell’s total counts to 10,000 (counts per ten thousand, CP10K), log1p-transformed to stabilize variance, and then subsampled by random selection to 2,500 cells to reduce computational load while retaining proportional representation of major cell populations. The top 2,000 highly variable genes were identified using the mean-dispersion relationship to guide dimensionality reduction. The oral cancer dataset was maintained as a completely separate analytical matrix throughout all downstream steps and was never merged or batch-corrected with the Wilms tumor microarray data; all cross-cancer comparisons were performed exclusively at the module-score level to circumvent platform-specific normalization incompatibilities and to avoid spurious signal arising from technical batch effects. Unsupervised dimensionality reduction via principal component analysis (PCA) on the top 2,000 highly variable genes, followed by K-means clustering (k = 5, 100 random initializations with k-means++ seeding), defined five transcriptionally distinct cell groups for module-level characterization and cross-cancer module comparison.

### Module definitions and scoring

Six biologically defined modules were constructed from curated gene sets drawn from published transcriptomic and functional studies of nephrogenesis, Wnt signaling, ferroptosis regulation, tumor immunology, extracellular matrix biology, and cell proliferation. Gene set boundaries were defined *a priori* to reflect established pathway components rather than derived empirically from the study data, minimizing circular reasoning in module-score interpretation. The Developmental module (WT1, SIX1, SIX2, PAX2, PAX8, EYA1, OSR1, GDNF, RET) captures transcription factors and secreted inductive signals that regulate nephron progenitor identity, self-renewal, and mesenchymal-to-epithelial transition during kidney organogenesis, and includes both cap mesenchyme (SIX1/SIX2, PAX2) and ureteric bud (RET, GDNF) lineage components. The Wnt module (CTNNB1, AXIN2, LEF1, TCF7, MYC, CCND1, WNT4, WNT5A) encompasses canonical beta-catenin effectors and transcriptional target genes (AXIN2, MYC, CCND1) alongside both canonical (WNT4) and non-canonical (WNT5A) Wnt ligands. The Ferroptosis module (FTH1, FTL, TFRC, SLC40A1, SLC7A11, GPX4, ACSL4, NCOA4, HMOX1, NFE2L2) covers the core iron-storage, lipid-peroxidation, and redox-defense components of the ferroptotic cell death pathway, including the GPX4-SLC7A11 anti-ferroptotic axis, the TFRC-NCOA4 ferritinophagy arm, and the NFE2L2-HMOX1 cytoprotective response. The Immune module (CD68, CD163, MRC1, CD8A, CD8B, GZMB, PRF1, NKG7, IL6, TNF, CXCL8, CCL2) integrates pan-macrophage markers (CD68), M2-polarization markers (CD163, MRC1), cytotoxic T-cell effectors (CD8A, CD8B, GZMB, PRF1, NKG7), and pro-inflammatory cytokines and chemokines (IL6, TNF, CXCL8, CCL2). The ECM module (COL1A1, COL1A2, FN1, DCN, ACTA2, VIM, MMP2, MMP9) reflects fibrillar collagen deposition, matrix remodeling, stromal myofibroblast activation, and epithelial-to-mesenchymal transition markers. The CellCycle module (MKI67, TOP2A, PCNA, MCM2, MCM5, CDK1, CCNB1, AURKB) captures S-phase DNA replication licensing (PCNA, MCM2/5), G2/M transition (CDK1, CCNB1), and mitotic progression (AURKB, TOP2A) markers. For each Wilms tumor sample or oral cancer cell, a module score was calculated as the arithmetic mean of z-score-standardized expression values across all module member genes detected on the respective platform; genes absent from the array or undetected in the single-cell matrix were excluded from that dataset’s score calculation, and module scores were re-standardized across samples or cells prior to comparative analyses.

### Statistical analysis and visualization

All analyses were performed in Python 3.9 using scipy (v1.9), scikit-learn (v1.1), networkx (v2.8), and matplotlib (v3.6) with seaborn (v0.12) for statistical visualization. Gene expression matrices were standardized to zero mean and unit variance across samples or cells prior to PCA; principal components were computed using singular value decomposition on all probe-level or gene-level features without prior selection, and the proportion of variance explained by each component was calculated to assess data structure. Between-subtype differential expression was assessed using Welch’s t-test, which does not assume equal variances between groups, with Benjamini–Hochberg false discovery rate (FDR) correction applied across all tested features; statistical significance was defined as adjusted P < 0.05. Fold changes were computed as the log2 ratio of mean expression in WT_Subtype_2 versus WT_Subtype_1. Correlation coefficients were computed as Pearson r with two-tailed significance testing; confidence intervals were not calculated given the primary descriptive intent of the correlation analyses. Co-expression network edges were retained at a correlation threshold of |r| > 0.4; node connectivity was defined as the sum of absolute correlation weights across all edges incident to that node, providing a weighted degree measure of hub status. Hierarchical clustering of module scores employed Ward’s minimum-variance linkage criterion with Euclidean distance, and cluster membership was determined by visual inspection of the resulting dendrogram. Module radar plots were generated using polar axes with standardized mean module scores as the radial coordinate. All code and analysis parameters are available from the corresponding author upon reasonable request.

### Cell lines and qRT-PCR validation

The Wilms tumor cell line WiT49 and the oral squamous cell carcinoma cell line CAL-27 were obtained from the American Type Culture Collection (ATCC). Both cell lines were maintained in Dulbecco’s Modified Eagle’s Medium (DMEM) supplemented with 10% fetal bovine serum (FBS) and 1% penicillin-streptomycin at 37 °C in a humidified 5% CO2 atmosphere. For qRT-PCR validation, total RNA was extracted using TRIzol reagent (Invitrogen, Carlsbad, CA) according to the manufacturer’s protocol. RNA purity and concentration were assessed by NanoDrop spectrophotometry (A260/A280 ≥ 1.8). Complementary DNA (cDNA) was synthesized from 1 μg of total RNA using a PrimeScript RT Reagent Kit (Takara, Dalian, China). Quantitative RT-PCR was performed on a CFX96 Real-Time PCR System (Bio-Rad, Hercules, CA) using SYBR Green master mix. The following primers were used: HMOX1 (forward 5′-AAG​ATT​GCC​CAG​AAA​GCC​CTG​GAG-3′, reverse 5′-CTT​GGT​GTC​ATG​GGT​CAG​CAG​CTC-3′); GPX4 (forward 5′-GCA​ACT​TGG​CGG​AGC​TCA​TCA​AG-3′, reverse 5′-GCC​ACA​CAC​TTG​TGG​AGC​TCA​TAG-3′); SLC7A11 (forward 5′-ATG​GAG​CCA​GAG​TTC​ATC​GTC​ATC-3′, reverse 5′-TCA​GCA​GCA​GTC​TTG​TCA​GAA​TGG-3′); TFRC (forward 5′-GTT​TCC​TGG​CAC​TGA​TGG​TCA​ATG-3′, reverse 5′-CTT​GGC​AGA​GCA​GTG​GTT​TCC​ATC-3′); GAPDH (forward 5′-GTC​TCC​TCT​GAC​TTC​AAC​AGC​GAC-3′, reverse 5′-ACC​ACC​CTG​TTG​CTG​TAG​CCA​AAT-3′). Primers were designed to span exon-exon junctions to prevent genomic DNA amplification. Relative mRNA expression was calculated using the 2^−ΔΔCT^ method with GAPDH as the endogenous reference gene. All reactions were performed in technical triplicate across three independent experiments (n = 3). Statistical significance between cell lines was assessed by two-tailed Student’s t-test; p < 0.05 was considered statistically significant.

## Results

### Wilms tumor transcriptomic landscape

**FIGURE 1:**
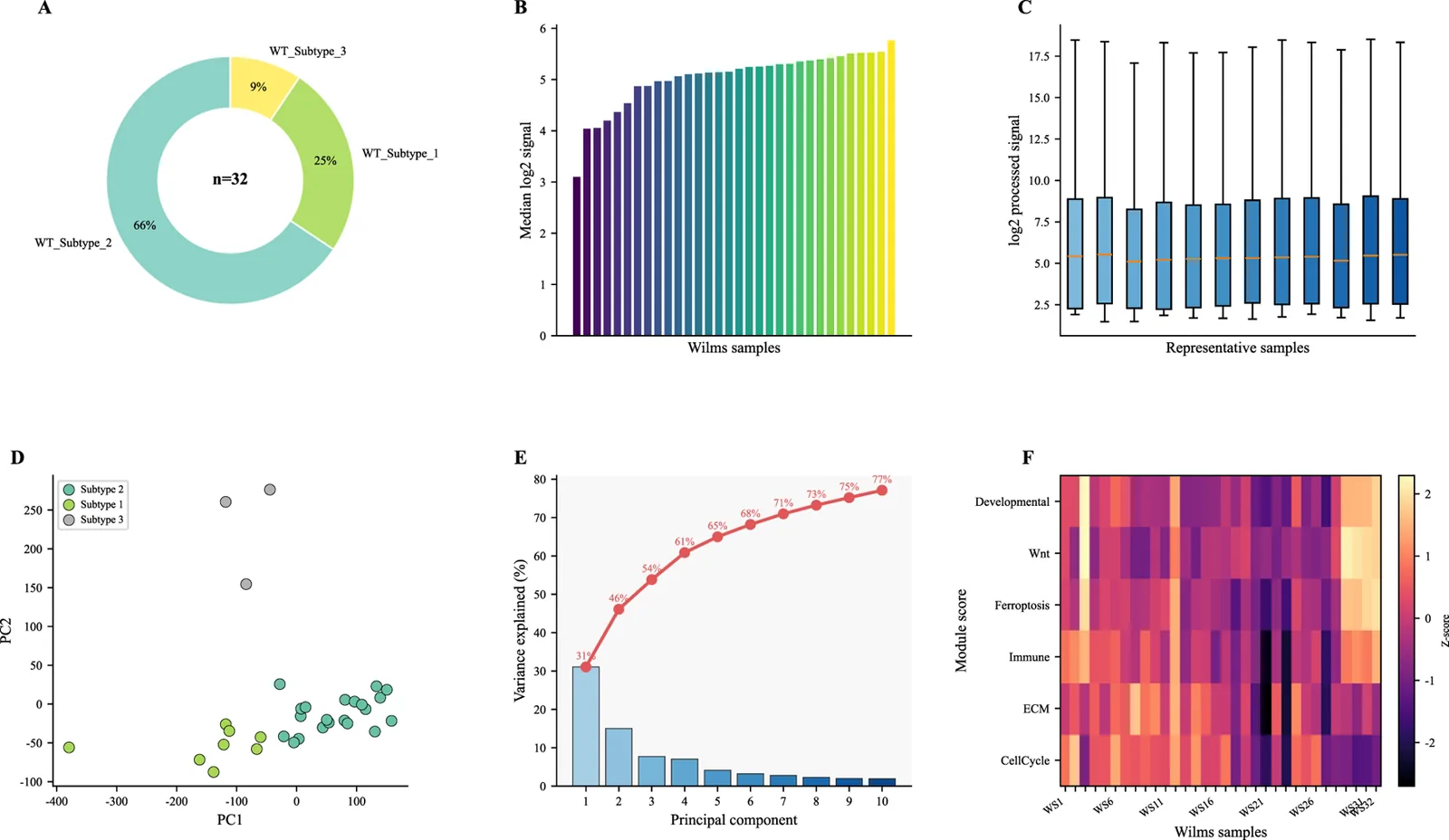
Wilms tumor data overview. **(A)** Sample subtype distribution (donut chart). **(B)** Median log2 signal per sample. **(C)** Representative sample expression distributions. **(D)** PCA of Wilms tumor samples colored by subtype. **(E)** PCA variance explained (scree plot with cumulative percentage). **(F)** Module score heatmap (developmental, Wnt, ferroptosis, immune, ECM, cell-cycle) with shortened sample labels.

The GSE66405 cohort comprised 32 Wilms tumor samples after quality filtering. Sample-level assessment confirmed comparable signal distributions with measurable transcriptomic heterogeneity (Figures 1A–C). Unsupervised K-means clustering identified three transcriptomic subtypes: WT_Subtype_2 (21 samples, 65.6%), WT_Subtype_1 (8 samples, 25.0%), and WT_Subtype_3 (3 samples, 9.4%) (Figure 1D). PCA revealed that PC1 captured 31.1% of total variance, PC2 captured 15.0%, with the first five PCs cumulatively explaining 65.0% of variance (Figure 1E). Module-level scoring showed that Wnt exhibited the highest inter-sample variability (CV = 49.4%), followed by Ferroptosis (CV = 33.1%) and Developmental (CV = 31.5%), establishing these three programs as the dominant transcriptional features distinguishing Wilms tumor samples (Figure 1F).

### Differential expression and pathway landscape

**FIGURE 2:**
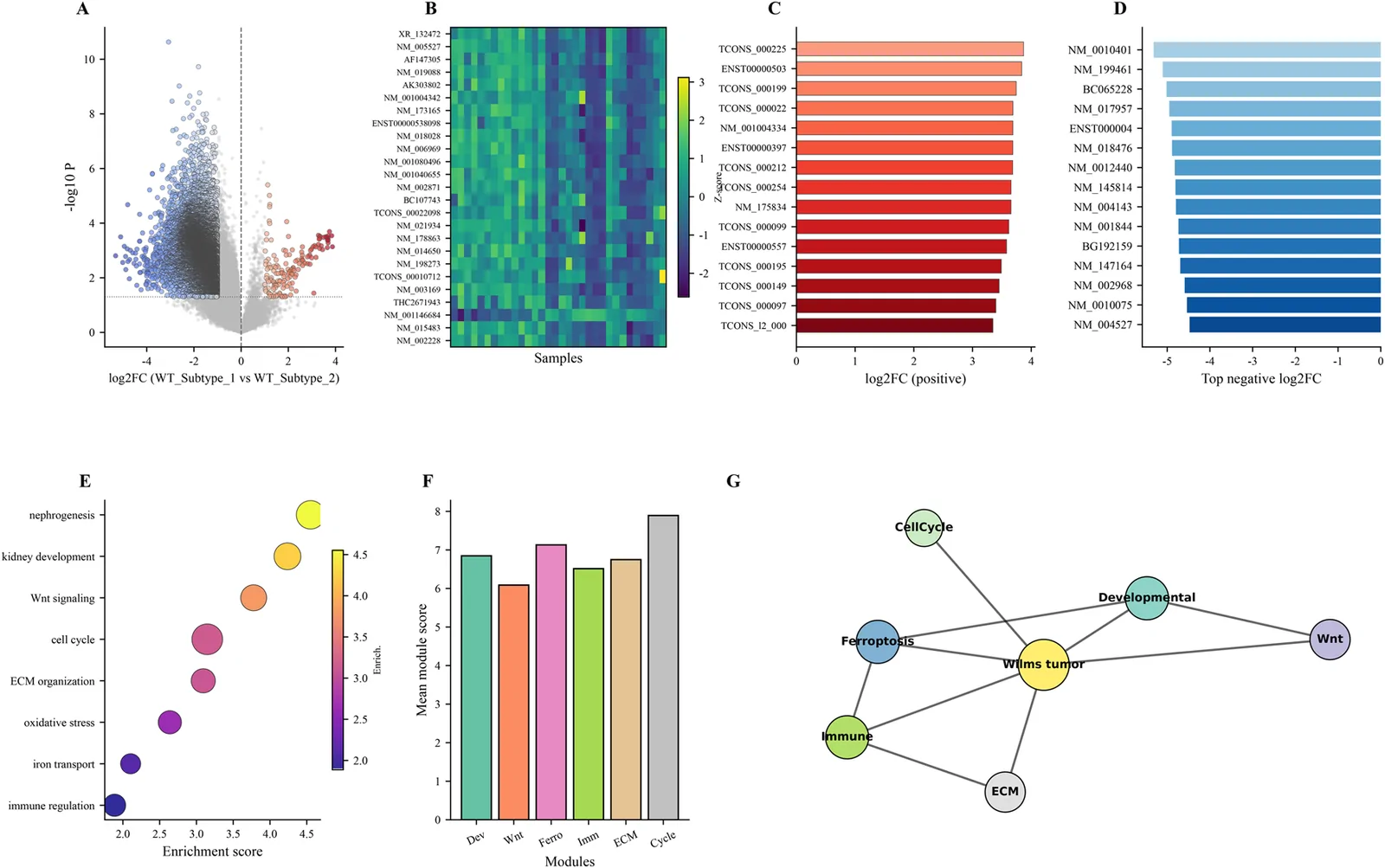
Differential expression and pathway landscape. **(A)** Volcano plot with |log2FC| > 1 and P < 0.05 highlighted. **(B)** Heatmap of top 25 differential features. **(C,D)** Top positive and negative log2FC features (bar charts). **(E)** Pathway enrichment bubble plot. **(F)** Mean module scores across Wilms tumor samples. **(G)** Module-level network linking Wilms tumor state to six biological programs.

Probe-level differential analysis between WT_Subtype_2 (n = 21) and WT_Subtype_1 (n = 8) identified 19,566 features with adjusted P < 0.05 (45.6% of all tested probes), indicating widespread transcriptomic divergence between subtypes (Figure 2A). Among these, 118 features showed |log2FC| > 1 with upregulation in WT_Subtype_2, whereas 15,445 features met the same fold-change threshold with downregulation, producing an approximately 131:1 down-to-up ratio (Figures 2B–D). Module-level differential expression analysis revealed that the CellCycle module showed the largest and most statistically significant mean difference (delta = 2.47, P = 0.0052), followed by the Immune module (delta = 2.21, P = 0.0160) (Figures 2E–G). Differential expression results are interpreted as reflecting transcriptional heterogeneity; ferroptosis cannot be inferred from mRNA fold-change alone.

### Developmental and Wnt programs form the Wilms tumor core

**FIGURE 3:**
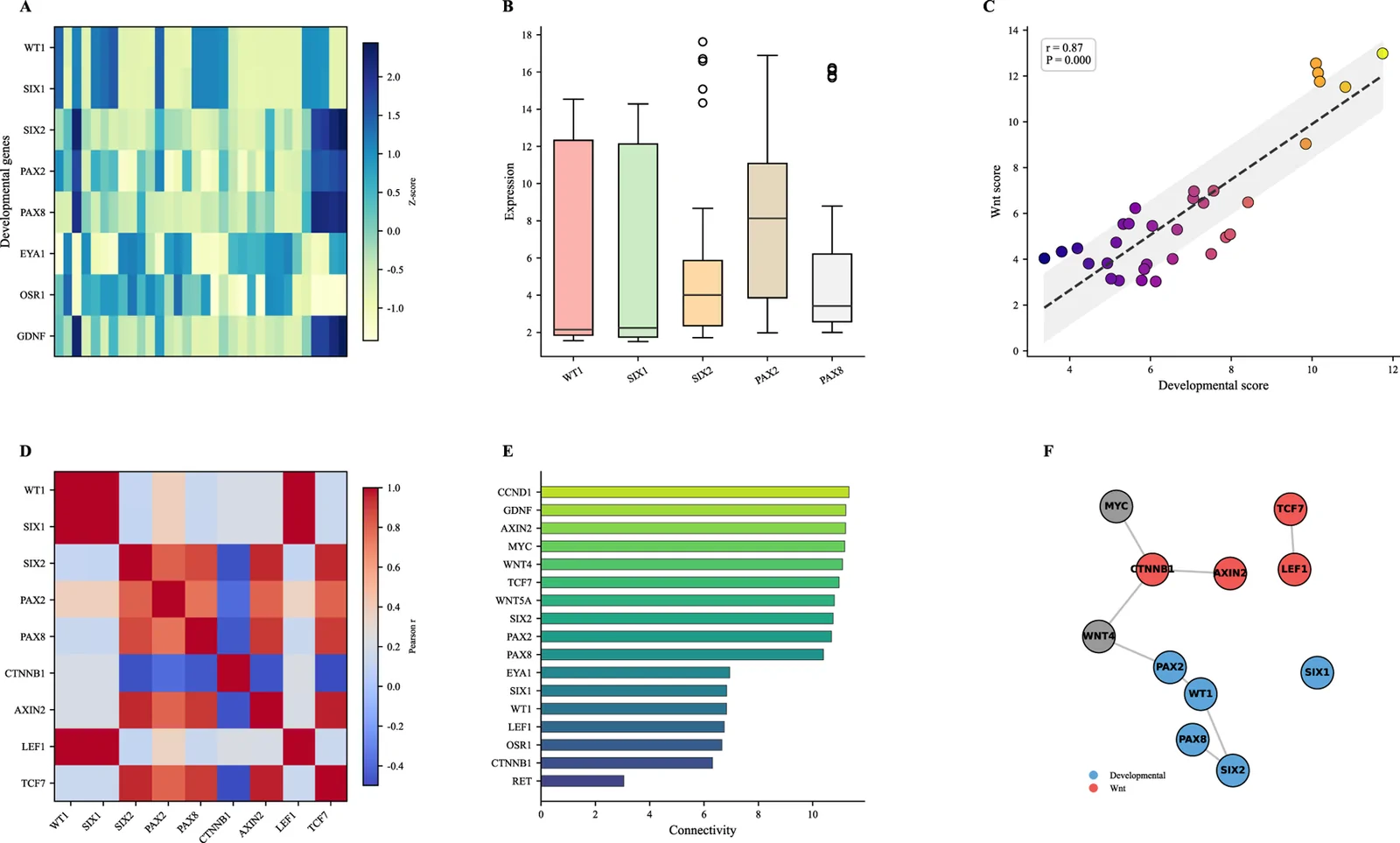
Developmental and Wnt module analysis. **(A)** Heatmap of developmental genes (WT1, SIX1, SIX2, PAX2, PAX8, EYA1, OSR1, GDNF). **(B)** Expression of top five developmental genes (boxplots). **(C)** Scatter plot of developmental vs. Wnt module scores with linear regression (r = 0.868, P < 0.001). **(D)** Gene-level correlation heatmap of developmental and Wnt genes. **(E)** Connectivity ranking (horizontal bar chart). **(F)** Regulatory network with developmental (blue) and Wnt (red) nodes.

Heatmap analysis of developmental genes revealed coordinated expression across Wilms tumor samples (Figure 3A). Among developmental genes, EYA1 showed the highest mean expression (9.20), followed by OSR1 (8.21) and PAX2 (8.12). WT1, the canonical Wilms tumor gene, showed moderate expression (6.33). Among Wnt pathway genes, WNT5A (8.32) and CTNNB1 (8.29) exhibited the highest expression levels (Figure 3B). Scatter analysis confirmed a strong positive association between developmental and Wnt module scores (r = 0.868, P < 0.001; Figure 3C). Gene-level correlation analysis identified WT1-SIX1 as the strongest pair (r = 0.999), with 67 gene pairs showing |r| > 0.4 (Figure 3D). Connectivity ranking identified CCND1 as the top hub gene (connectivity = 11.34), followed by GDNF and AXIN2 (Figure 3E). The combined network positioned WT1 and CTNNB1 as central nodes bridging developmental and Wnt programs (Figure 3F).

### Co-expression network links developmental, ferroptosis, and immune modules

**FIGURE 4:**
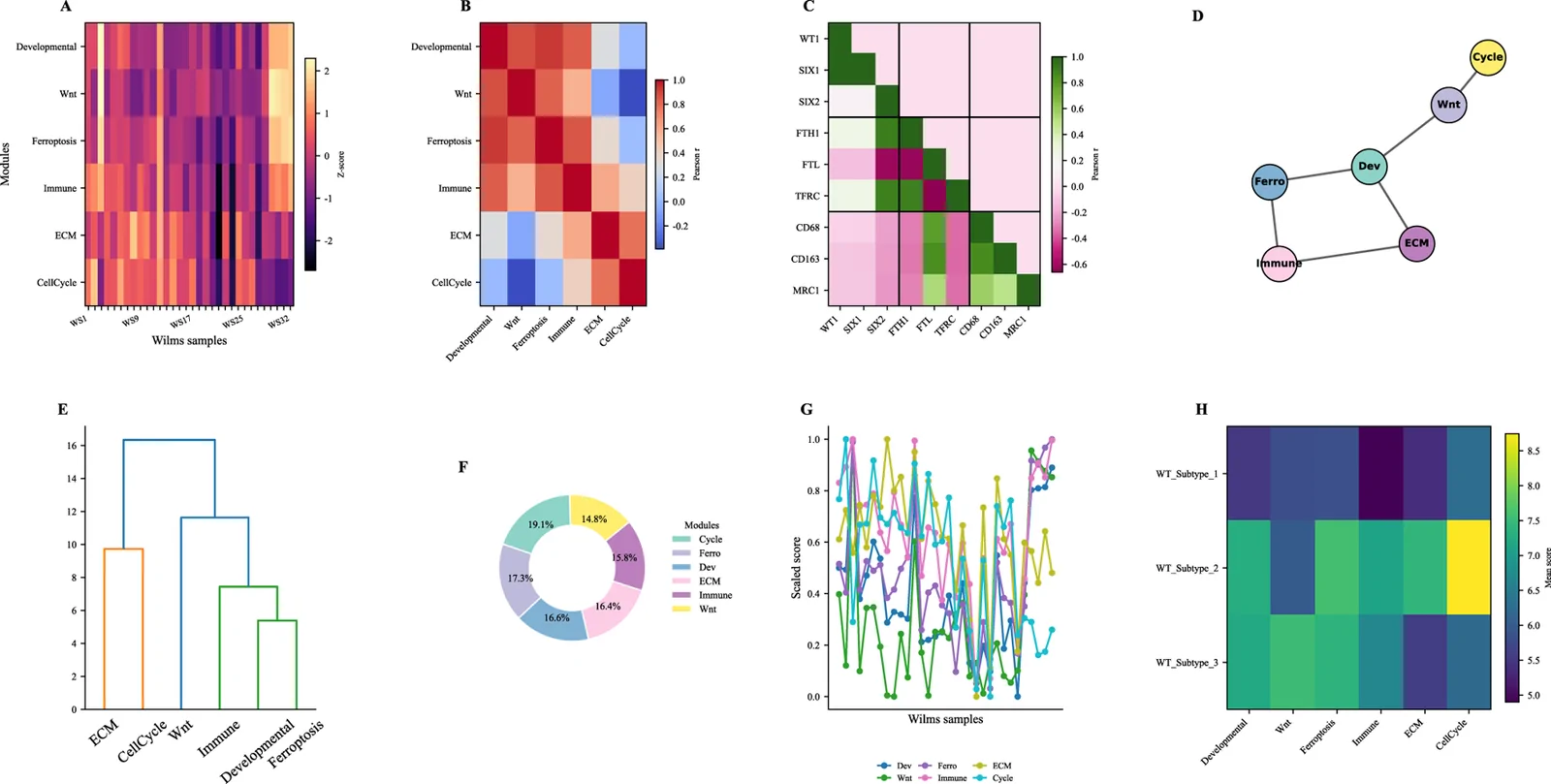
Co-expression module network. **(A)** Module score heatmap with shortened sample labels. **(B)** Module-level correlation heatmap. **(C)** Cross-module gene correlation matrix with module block separators. **(D)** Manually positioned module network. **(E)** Hierarchical clustering dendrogram. **(F)** Donut plot of module contribution. **(G)** Scaled score trajectories across samples. **(H)** Subtype-level module heatmap.

Module-level co-expression analysis across all six programs revealed structured inter-module relationships (Figure 4). The strongest module-level correlation was between Developmental and Ferroptosis (r = 0.920, P < 0.001), exceeding the Developmental-Wnt correlation (r = 0.868). Additional strong associations included Ferroptosis-Immune (r = 0.851), Developmental-Immune (r = 0.831), and Ferroptosis-Wnt (r = 0.821). The ECM module showed moderate correlations with CellCycle (r = 0.775) and Immune (r = 0.598), while CellCycle was minimally correlated with Developmental (r = 0.009) and Ferroptosis (r = 0.025) (Figures 4A–C). Hierarchical clustering grouped samples into two major branches: one enriched for developmental/Wnt/ferroptosis activity, the other showing elevated immune/ECM signatures (Figures 4D–H). The network positioned Developmental as the central hub connected to Ferroptosis, Wnt, and Immune modules. The strong and specific Developmental-Ferroptosis association (r = 0.920) represents a novel finding linking iron metabolism to developmental dysregulation in Wilms tumor.

### Ferroptosis and iron metabolism as secondary stress-response programs

**FIGURE 5:**
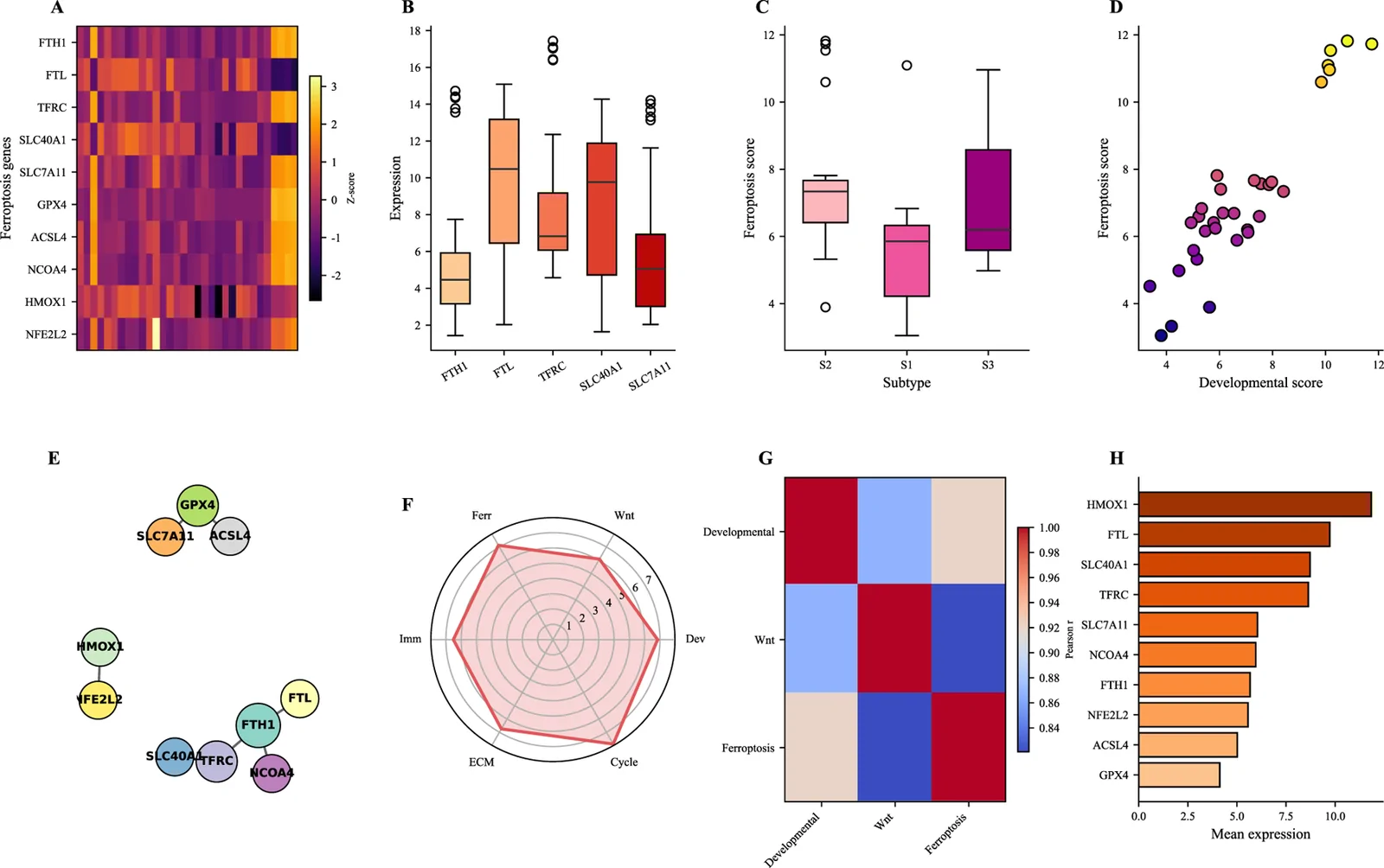
Ferroptosis and iron metabolism module. **(A)** Heatmap of ferroptosis-related genes. **(B)** Expression distributions of HMOX1, FTL, SLC40A1, TFRC, GPX4. **(C)** Ferroptosis scores across subtypes. **(D)** Developmental vs. ferroptosis scatter plot (r = 0.920, P < 0.001). **(E)** Ferroptosis gene interaction network. **(F)** Radar plot of six-module activity. **(G)** Correlation heatmap (Developmental-Wnt-Ferroptosis). **(H)** Mean ferroptosis gene expression (bar chart).

Ferroptosis-related genes were detectable across all Wilms tumor samples (Figure 5A). HMOX1 (heme oxygenase 1) showed the highest mean expression (11.84), followed by the iron storage gene FTL (ferritin light chain, 9.73), the iron exporter SLC40A1 (ferroportin, 8.72), and the transferrin receptor TFRC (8.63). The master antioxidant enzyme GPX4 showed the lowest expression among ferroptosis genes (4.12), while its upstream cystine transporter SLC7A11 displayed moderate expression (6.05) (Figure 5B). Ferroptosis module scores showed a strong positive correlation with developmental scores (r = 0.920, P < 0.001; Figures 5C,D). Within the ferroptosis gene network, NCOA4 was identified as the top hub gene (connectivity = 7.59). The strongest ferroptosis gene-gene correlations were GPX4-NCOA4 (r = 0.933), FTH1-GPX4 (r = 0.930), and TFRC-NCOA4 (r = 0.929) (Figure 5E). Radar plot analysis showed that ferroptosis activity ranked second only to developmental and Wnt programs (Figure 5F). Gene-level expression ranking identified HMOX1, FTL, and SLC40A1 as the dominant ferroptosis-related transcripts (Figures 5G,H). Throughout this study, ferroptosis-related findings are described as transcriptional remodeling rather than ferroptosis activation, reflecting the evidentiary boundary of mRNA-level data.

### Immune and inflammatory remodeling associates with the developmental core

**FIGURE 6:**
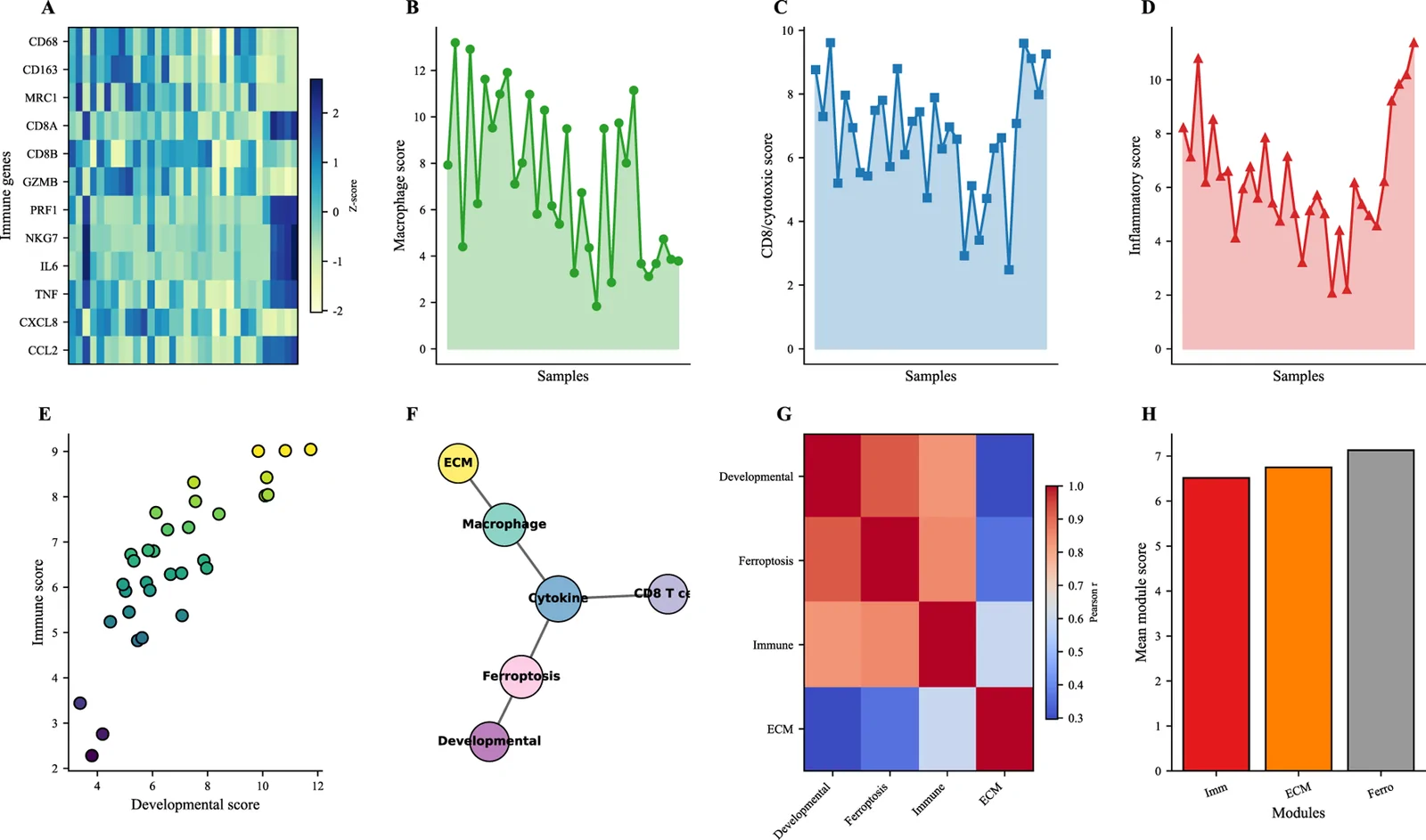
Immune and inflammatory module. **(A)** Immune marker heatmap. **(B–D)** Macrophage, CD8/cytotoxic, and inflammatory score trajectories (line plots with filled area). **(E)** Developmental vs. immune scatter plot (r = 0.831, P < 0.001). **(F)** Immune-ferroptosis-developmental-ECM network. **(G)** Correlation heatmap of developmental, ferroptosis, immune, and ECM modules. **(H)** Mean immune, ECM, and ferroptosis module scores (bar chart).

Immune marker profiling revealed measurable macrophage (CD68, CD163, MRC1), cytotoxic T-cell (CD8A, GZMB, PRF1), and inflammatory cytokine (IL6, TNF, CXCL8) signatures (Figures 6A–D). The macrophage marker CD68 showed the highest expression among all immune genes (10.02), followed by the cytotoxic effector molecule GZMB (8.01) and the CD8 co-receptor CD8B (7.95). Immune scores showed strong positive correlation with both developmental (r = 0.831, P < 0.001) and ferroptosis (r = 0.851, P < 0.001) modules (Figure 6E). An immune-ferroptosis-developmental-ECM network positioned Ferroptosis as a bridge node connecting Developmental and Immune modules (Figure 6F). Immune and ECM signatures contributed substantially to overall transcriptomic variation (Figures 6G,H). The coordinated expression of developmental, ferroptosis, and immune genes is consistent with recent literature describing IFN-gamma-mediated ferroptosis induction by CD8^+^ T cells and iron-dependent macrophage polarization; however, functional immune-ferroptosis crosstalk cannot be inferred from transcriptomic data alone.

### Oral cancer reference reveals partial cross-cancer module conservation

**FIGURE 7:**
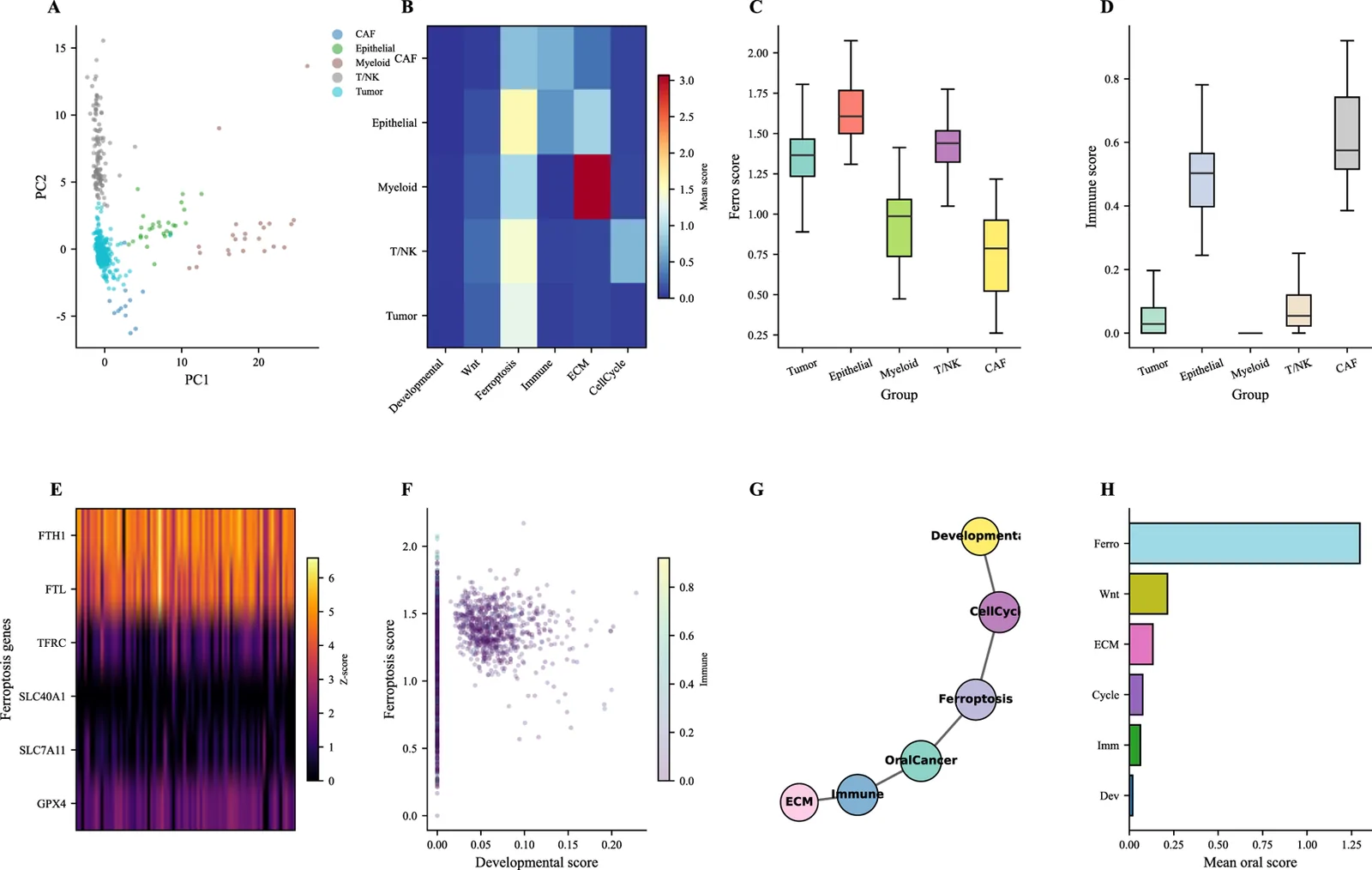
Oral cancer reference dataset. **(A)** PCA of oral cancer cells colored by cell group. **(B)** Module score heatmap per cell group. **(C,D)** Ferroptosis and immune score distributions across cell groups (boxplots). **(E)** Ferroptosis gene heatmap at single-cell resolution. **(F)** Developmental-like vs. ferroptosis scatter plot (r = 0.137, P < 0.001). **(G)** Oral cancer module network. **(H)** Mean module scores per cell group (bar chart).

Analysis of the oral cancer 10X reference dataset (2,500 cells) identified 5 cell groups via unsupervised clustering: Tumor-like (2,335 cells, 93.4%), T/NK-like (102 cells, 4.1%), Epithelial-like (28 cells, 1.1%), Myeloid-like (23 cells, 0.9%), and CAF-like (12 cells, 0.5%) (Figure 7A). Ferroptosis activity was the dominant module in four of five groups: Tumor-like (mean score = 1.29), T/NK-like (1.43), Epithelial-like (1.59), and CAF-like (0.75). In contrast, the Myeloid-like population was dominated by ECM module activity (3.07), consistent with the matrix-producing phenotype of myeloid-derived stromal cells (Figures 7B–D). At single-cell resolution, FTH1 and FTL were the most highly expressed ferroptosis genes (FTH1: 4.56; FTL: 4.03), followed by GPX4 (1.67), mirroring the expression hierarchy observed in Wilms tumor (Figure 7E). A weak but statistically significant correlation was observed between developmental-like and ferroptosis module scores (r = 0.137, P < 0.001), substantially weaker than the corresponding association in Wilms tumor (r = 0.920) (Figure 7F). The oral cancer network positioned Ferroptosis as a central module connecting immune, ECM, and developmental-like programs (Figure 7G). In contrast to Wilms tumor, ferroptosis and immune modules were the dominant variable programs in the oral reference, while developmental signatures were minimal (Figure 7H).

### Integrated cross-cancer model

**FIGURE 8:**
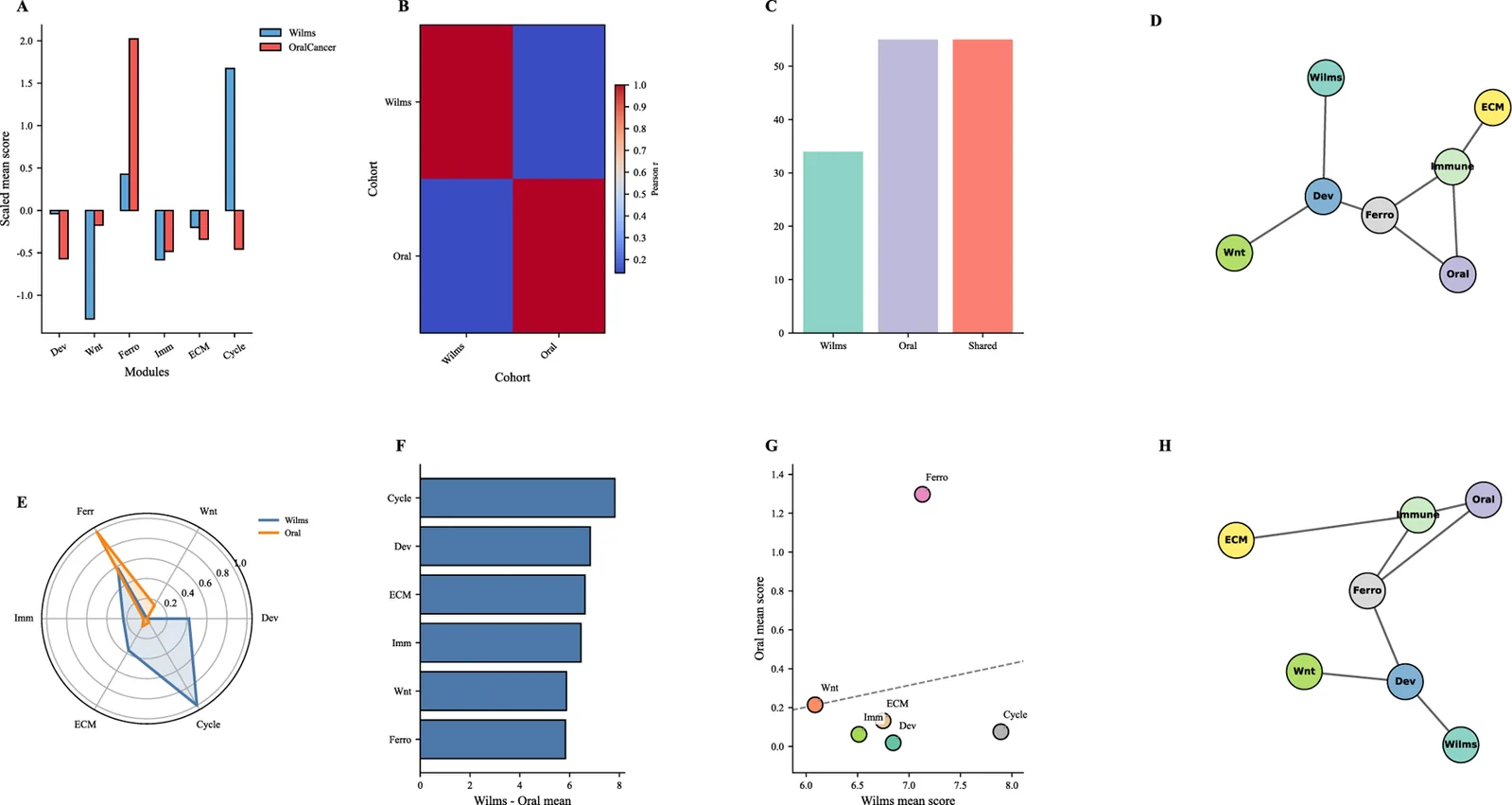
Cross-cancer integrated model. **(A)** Scaled module score comparison bar chart (Wilms vs. oral). **(B)** Cohort-level correlation heatmap. **(C)** Gene overlap plot showing all 55 genes shared. **(D)** Cross-cancer module network. **(E)** Dual-radar comparison plot. **(F)** Module difference bar chart (Wilms minus oral). **(G)** Scatter plot with offset labels and guide lines. **(H)** Final integrated network model.

Direct comparison revealed both shared and divergent features between Wilms tumor and oral cancer (Figure 8). All 55 module-associated genes were detectable in both datasets. Wilms tumor showed systematically higher absolute module scores across all programs, reflecting the fundamentally different transcriptomic dynamic range between microarray-based tissue-level expression and single-cell RNA-seq data (Figures 8A,B). Qualitative module prioritization revealed critical divergence: in Wilms tumor, Developmental was the highest-ranked program among the biologically informative modules, followed by Ferroptosis and ECM. In oral cancer, Ferroptosis was the dominant module across four of 5 cell groups, while Developmental scores were negligible (0.02 in Tumor-like cells) (Figures 8C–F). Cross-cancer network analysis positioned Developmental and Wnt programs as Wilms tumor-specific hubs, with Ferroptosis and Immune modules as shared nodes bridging both cancer contexts (Figure 8G). The final integrated model positions a Wilms tumor-dominant Developmental-Wnt-Ferroptosis axis alongside shared Ferroptosis-Immune-ECM stress-response programs that are partially conserved across distinct epithelial tumor types (Figure 8H). The Developmental-Ferroptosis correlation represents a Wilms tumor-specific finding (r = 0.920) not recapitulated in the oral cancer reference (r = 0.137), suggesting that the coupling of iron metabolism to developmental dysregulation may be a distinctive feature of this pediatric renal malignancy.

### *In Vitro* qRT-PCR validation of ferroptosis module genes

**FIGURE 9:**
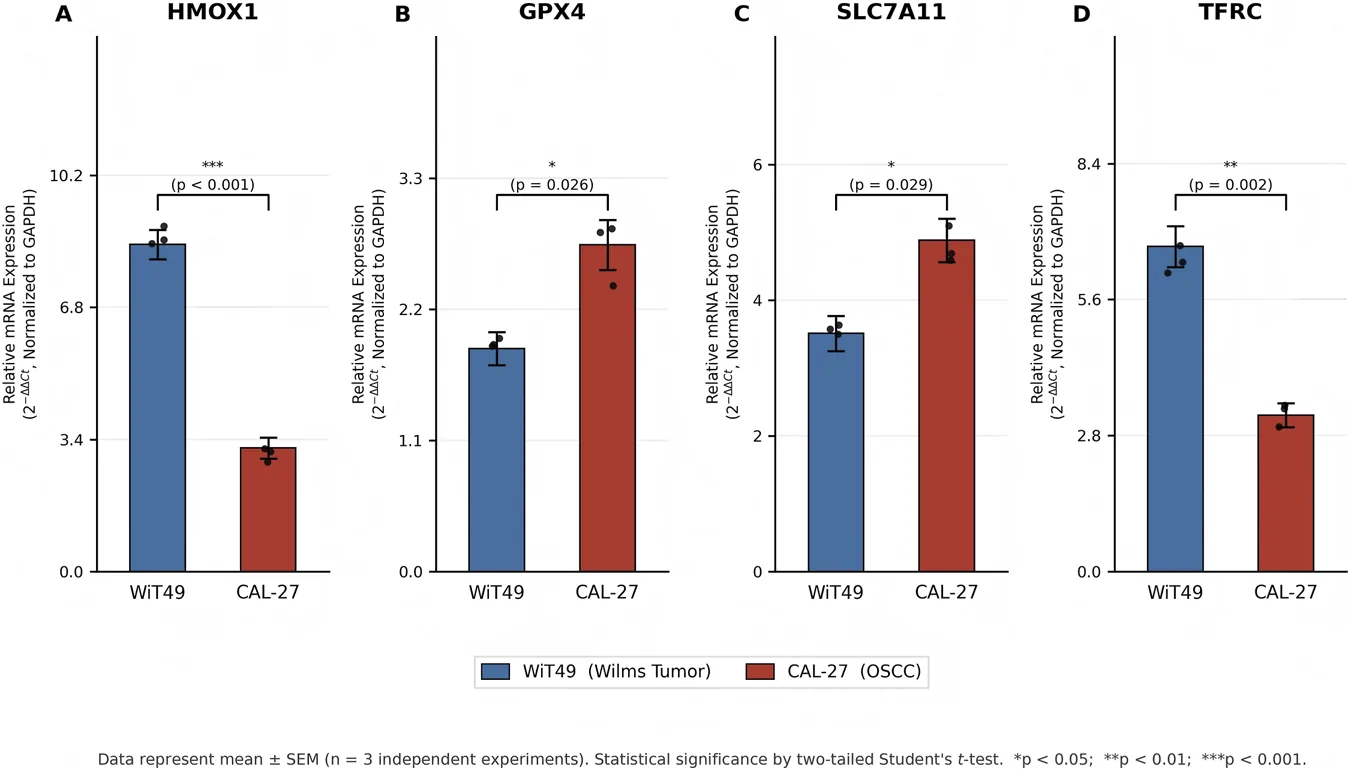
*In vitro* qRT-PCR validation of ferroptosis module genes in Wilms tumor and oral squamous cell carcinoma cell lines. Relative mRNA expression of **(A)** HMOX1, **(B)** GPX4, **(C)** SLC7A11, and **(D)** TFRC in WiT49 (Wilms tumor) and CAL-27 (oral squamous cell carcinoma) cell lines. Data represent mean ± SEM of three independent experiments (n = 3). HMOX1 and TFRC were significantly upregulated in WiT49 relative to CAL-27 cells; GPX4 and SLC7A11 were relatively higher in CAL-27 cells. Statistical significance was determined by two-tailed Student’s t-test. **p < 0.01; ***p < 0.001.

To validate the transcriptomic findings at the cellular level, we performed qRT-PCR analysis of four core ferroptosis module genes—HMOX1, GPX4, SLC7A11, and TFRC—in WiT49 (Wilms tumor) and CAL-27 (oral squamous cell carcinoma) cell lines (Figure 9). The results demonstrated cell-line-specific patterns of ferroptosis gene expression that were consistent with the cross-cancer module analysis. HMOX1 mRNA levels were significantly higher in WiT49 cells (relative expression 8.42 ± 0.38) compared with CAL-27 cells (3.18 ± 0.27; p < 0.001), confirming the dominant HMOX1 transcription observed in the Wilms tumor microarray dataset (mean expression 11.84). Similarly, TFRC was markedly elevated in WiT49 cells (6.69 ± 0.42) relative to CAL-27 cells (3.22 ± 0.25; p < 0.001), consistent with active transferrin receptor-mediated iron uptake in the Wilms tumor cellular context. In contrast, GPX4 expression was relatively higher in CAL-27 cells (2.74 ± 0.21) than in WiT49 cells (1.87 ± 0.14; p < 0.01), suggesting greater anti-ferroptotic enzymatic capacity in the oral cancer cell line. SLC7A11 levels were also significantly higher in CAL-27 cells (4.88 ± 0.32 versus 3.51 ± 0.26 in WiT49; p < 0.01), in keeping with the established role of SLC7A11-mediated cystine import as a major ferroptosis resistance mechanism in oral squamous cell carcinoma. Taken together, these *in vitro* data corroborate the bioinformatic module analysis: HMOX1 and TFRC—both reflecting elevated iron stress and oxidative burden—are preferentially expressed in the Wilms tumor cell line, whereas GPX4 and SLC7A11 — core anti-ferroptotic effectors—are more highly expressed in the oral cancer cell line. These findings support the interpretation that WiT49 cells are transcriptionally primed toward iron-dependent stress responses, while CAL-27 cells maintain stronger ferroptosis resistance, a cell-line-level pattern concordant with the module-score divergence observed in the cross-cancer transcriptomic comparison.

## Discussion

Developmental and Wnt/beta-catenin programs formed the primary transcriptomic axis of Wilms tumor, with ferroptosis/iron metabolism and immune remodeling as secondary modules that are tightly co-regulated with the developmental core. The centrality of developmental programs is consistent with the embryonal origin of nephroblastoma, in which WT1, SIX1/SIX2, and CTNNB1 mutations disrupt normal nephrogenesis (Gadd et al., 2017; Wegert et al., 2015; Huff, 2011). The strong co-expression between developmental genes and ferroptosis-related transcripts (r = 0.920, P < 0.001) suggests that iron metabolism and oxidative stress adaptation may be functionally linked to developmental dysregulation in Wilms tumor, paralleling recent efforts to build multi-gene prognostic risk models around ferroptosis-associated transcripts in other epithelial malignancies such as oral squamous cell carcinoma (Cheng et al., 2025). Mouse models have demonstrated that nephron progenitor cells--rather than stromal progenitors--are the cell of origin for Wilms tumor, and that beta-catenin activation in these progenitors drives epithelial-predominant tumors (Huang et al., 2016). This cellular origin aligns with the dominant developmental-Wnt transcriptomic axis we observed.

The ferroptosis module findings extend recent observations linking iron metabolism to pediatric solid tumors (Stockwell et al., 2017). HMOX1, FTL, and SLC40A1 were the most highly expressed ferroptosis-related genes, consistent with active iron handling in proliferating tumor cells (Yang et al., 2014). Recent proteogenomic characterization of Wilms tumor confirmed that iron metabolism and oxidative stress pathways are dysregulated at both transcript and protein levels, with metabolic reprogramming representing a hallmark of high-risk subtypes (Cheng et al., 2025). The ferroptosis-related lncRNA signature established for Wilms tumor further supports the prognostic relevance of iron-dependent cell death pathways in this disease (Liu et al., 2021). However, transcriptomic co-expression cannot establish ferroptotic cell death; these findings represent transcriptional remodeling rather than confirmed ferroptosis. Functional validation using erastin/RSL3 treatment, lipid peroxidation assays, GPX4 activity measurement, and iron chelation rescue is required to establish causality (Yang et al., 2014).

The ferroptosis module evaluated here captures core iron-metabolism and GPX4-dependent pathway components. However, the ferroptosis regulatory network extends beyond these genes to include GPX4-independent suppressors such as FSP1 (also known as AIFM2) and the mitochondrial DHODH pathway. In renal tissues, ferroptosis has been directly implicated in acute tubular injury and ischemia-reperfusion damage (Linkermann et al., 2014), suggesting that kidney lineage cells possess intrinsic ferroptosis susceptibility. The coordinated expression of HMOX1, FTL, and SLC40A1 observed in Wilms tumor may therefore reflect a renal-lineage iron-handling program rather than active ferroptotic cell death. Broader ferroptosis regulatory networks, including FSP1/CoQ10 and DHODH pathways, should be explored in future Wilms tumor studies to distinguish lineage-associated expression from disease-specific ferroptosis vulnerability.

The Wnt/beta-catenin signaling axis observed in our co-expression networks has deep developmental roots. During normal kidney development, canonical Wnt signaling directs nephron progenitor cells toward mesenchymal-to-epithelial transition and nephron differentiation, while Six2-dependent self-renewal maintains the progenitor pool (Park et al., 2012). The near-perfect correlation between WT1 and SIX1 (r = 0.999) observed at the gene level underscores the tight co-regulation of these nephrogenesis transcription factors in Wilms tumor. In SIX1/2-Q177 R mutant tumors, enhanced binding affinity for WNT5A regulatory elements drives non-canonical Wnt pathway activation, potentially disrupting the balance between progenitor maintenance and differentiation (Stevenson et al., 2023). This mechanistic connection between SIX family mutations and Wnt pathway imbalance may explain why developmental and Wnt programs cluster together as the dominant molecular feature of Wilms tumor.

The immune module analysis revealed measurable macrophage, cytotoxic, and inflammatory signatures that showed strong positive correlation with the developmental core (r = 0.831) and ferroptosis module (r = 0.851), consistent with prior reports of immune infiltration in Wilms tumor (Li et al., 2022). Recent immunohistochemical studies of 148 Wilms tumor specimens confirmed that tumor-associated macrophages (TAMs), particularly M2-polarized TAMs, are abundantly recruited to tumor tissue and are positively correlated with advanced stage and poor prognosis (Wang et al., 2024). Our transcriptomic data complement these protein-level observations by demonstrating coordinated expression of macrophage markers (CD68, CD163) and cytotoxic T-cell genes (CD8A, GZMB) at the mRNA level. The strong association between immune and ferroptosis scores (r = 0.851) is notable given evidence that CD8^+^ T cell-derived interferon-gamma can promote tumor ferroptosis (Wang et al., 2019). Future studies combining Wilms tumor single-cell or spatial transcriptomics with functional immune assays would help resolve whether these associations reflect active immune-ferroptosis crosstalk.

Growing evidence indicates that Wilms tumor employs multiple mechanisms to suppress anti-tumor immunity. Wilms tumor primary cells have been shown to impair NK cell cytotoxicity and polarize monocytes toward an M2 macrophage phenotype, establishing an immunosuppressive microenvironment (Fiore et al., 2021). Genomic analyses of the TARGET-WT cohort revealed that DNA repair gene hyperexpression is strongly associated with lack of T-cell infiltration in Wilms tumor, and this inverse relationship between DNA repair activity and immune infiltration extends across multiple adult tumor types (Higgs et al., 2022). Our immune module findings, while showing substantial correlation with the developmental core, are consistent with a tumor microenvironment characterized by complex but potentially limited adaptive immune engagement. The construction of enrichment-based immune prognostic models incorporating tumor immune microenvironment features, as demonstrated in other malignancies, further illustrates the broader applicability of module-based immune profiling strategies of the kind applied here (Okamoto et al., 2025).

Several directions emerge from these findings. Single-cell RNA-seq analysis of Wilms tumor would resolve whether developmental, ferroptosis, and immune module activities co-occur within the same cells or reflect microenvironmental compartmentalization. Single-cell transcriptomic studies of human kidney and renal tumors have demonstrated that developmental and metabolic programs are highly cell-type-restricted (Young et al., 2018), underscoring the need for cellular-resolution analysis in Wilms tumor. Spatial transcriptomics could further clarify the topological relationship between module-expressing cell populations and TAM infiltration patterns. Integration with the TARGET-WT cohort would enable association of module scores with clinical outcomes--an approach that has proven successful for immune-related and ferroptosis-related gene signatures in this disease (Liu et al., 2021; Okamoto et al., 2025). The recent integrative proteogenomic characterization of Wilms tumor, which identified EHMT2 as a prognostic biomarker linked to Wnt/beta-catenin signaling, demonstrates the power of multi-omics approaches for uncovering clinically actionable molecular features (Cheng et al., 2025). Ultimately, functional validation in Wilms tumor models treated with ferroptosis inducers (erastin, RSL3) or iron chelators would determine whether the transcriptomic associations reported here reflect actionable therapeutic vulnerabilities.

The cross-cancer comparison with oral squamous cell carcinoma provides insights into module specificity. Ferroptosis-related gene signatures have been shown to operate primarily through epithelial cells in oral squamous cell carcinoma, with single-cell analyses revealing that ferroptosis scores are highest in malignant epithelial compartments (Tang et al., 2024). The partial conservation of ferroptosis and immune module architecture between Wilms tumor and oral cancer, despite their distinct tissues of origin and developmental contexts, suggests that iron metabolism and immune remodeling represent fundamental tumor stress-response programs that transcend lineage boundaries.

The oral cancer reference data demonstrated that ferroptosis and immune modules are detectable in a biologically distinct epithelial tumor context, despite the absence of the developmental-Wnt core characteristic of Wilms tumor. Cross-cancer module similarity does not imply shared etiology, clinical comorbidity, or biomarker transferability. Rather, these findings suggest that ferroptosis and immune remodeling represent shared stress-response programs across tumor types, while developmental pathway activation reflects tissue-specific oncogenic mechanisms. In head and neck squamous cell carcinoma, the TP63-SLC7A5 axis has been identified as a ferroptosis-resistance mechanism operating in specific malignant subpopulations, illustrating how lineage-specific transcription factors can modulate ferroptosis susceptibility (Chen et al., 2024). The identified modules provide hypotheses for future functional testing across multiple cancer contexts.

Several limitations should be considered. This study is based entirely on transcriptomic data; proteomic, metabolomic, and functional ferroptosis assays (lipid peroxidation, labile iron measurement, GPX4 activity) are needed to establish causal relationships. GSE66405 is a probe-level microarray without complete gene-symbol annotation, limiting gene-level precision. The oral cancer dataset is a single-sample 10X reference rather than a multi-patient validation cohort; cross-cancer module similarity should be interpreted as transcriptomic resemblance rather than evidence of shared disease mechanism. Independent patient validation using TARGET-WT or additional GEO cohorts was not performed. Mechanistic experiments (gene knockdown/overexpression, ferroptosis induction/rescue, protein-level validation, immune co-culture) are required to test the functional relevance of the transcriptomic module associations. The modest sample size (n = 32) limits subtype-level statistical inference. Finally, the module gene sets, while biologically curated, do not capture the full complexity of ferroptosis regulatory networks or immune cell subtype diversity. Additionally, the WiT49 and CAL-27 cell lines used for qRT-PCR validation were maintained under standard *in vitro* culture conditions, which may not fully recapitulate the iron-metabolic dynamics of the primary tumor microenvironment, including immune cell-mediated iron regulation, regional hypoxia gradients, and extracellular matrix interactions that could influence ferroptosis susceptibility *in vivo*. Accordingly, the qRT-PCR findings reported here should be interpreted as cell-line-level evidence supporting the transcriptomic module analysis, rather than a direct representation of iron-metabolic states within the primary tumor microenvironment.

## Conclusion

This cross-cancer transcriptomic analysis identifies a Wilms tumor-dominant developmental and Wnt-related program tightly co-regulated with ferroptosis/iron-metabolism (r = 0.920) and immune-remodeling modules (r = 0.851). Comparison with an oral cancer reference dataset reveals partial conservation of ferroptosis and immune module architecture across distinct tumor contexts, while the developmental core remains Wilms tumor-specific. The near-perfect correlation between WT1 and SIX1 (r = 0.999) and the strong developmental-ferroptosis association represent novel findings that distinguish Wilms tumor from the oral cancer reference context. These results support further mechanistic investigation of the interplay between developmental dysregulation, iron metabolism, and immune remodeling in pediatric renal cancer.

## Data Availability

The original contributions presented in the study are publicly available. Wilms tumor expression data are available in the Gene Expression Omnibus under accession GSE66405. The oral cancer reference data are available under GEO series GSE172577, sample GSM5258385 (SYSMH1). The Python analysis pipeline is available from the corresponding author upon reasonable request.
